# Novel expression hosts for complex secondary metabolite megasynthetases: Production of myxochromide in the thermopilic isolate *Corallococcus macrosporus *GT-2

**DOI:** 10.1186/1475-2859-8-1

**Published:** 2009-01-06

**Authors:** Olena Perlova, Klaus Gerth, Silvia Kuhlmann, Youming Zhang, Rolf Müller

**Affiliations:** 1 Institut für Pharmazeutische Biotechnologie, Universität des Saarlandes, Postfach 15 11 50, D-66041 Saarbrücken, Germany; 2 Helmholtz-Zentrum für Infektionsforschung GmbH, MWIS, Inhoffenstraße 7, D-38124 Braunschweig, Germany; 3 Gene Bridges GmbH, BioInnovationsZentrum, Tatzberg 47, 01307 Dresden, Germany

## Abstract

Although many secondary metabolites with diverse biological activities have been isolated from myxobacteria, most strains of these biotechnologically important gliding prokaryotes remain difficult to handle genetically. In this study we describe the new fast growing myxobacterial thermophilic isolate GT-2 as a heterologous host for the expression of natural product biosynthetic pathways isolated from other myxobacteria. According to the results of sequence analysis of the 16S rDNA, this moderately thermophilic isolate is closely related to *Corallococcus macrosporus *and was therefore named *C. macrosporus *GT-*2*. Fast growth of moderately thermophilic strains results in shorter fermentation and generation times, aspects which are of significant interest for molecular biological work as well as production of secondary metabolites. Development of a genetic manipulation system allowed the introduction of the complete myxochromide biosynthetic gene cluster, located on a transposable fragment, into the chromosome of GT-2. Genetic engineering of the biosynthetic gene cluster by promoter exchange leads to much higher production of myxochromides in the heterologous host *C. macrosporus *GT-2 in comparison to the original producer *Stigmatella aurantiaca *and to the previously described heterologous host *Pseudomonas putida *(600 mg/L versus 8 mg/L and 40 mg/L, respectively).

## Background

Although the world wide demand for novel anti-infectious agents is becoming more and more pressing, several pharmaceutical companies withdrew from "new antibiotic" research because of the long development times and the high financial risk. At the same time antibiotic resistance of numerous pathogenic organisms is increasing quickly. In addition, globalization and changes in socio-economic conditions increase the risk of a spread of currently unknown infectious microorganisms and agents [1]. During the last two decades, myxobacteria became widely known as valuable producers of secondary metabolites exhibiting various biological activities [2,3]. However, the optimization of production of the already known metabolites with promising biological activities like epothilones [4] or tubulysins [5,6] remains a challenging task. Myxobacteria are ubiquitous microorganisms which live on rotting plant material, animal dung and in soils worldwide [7-9]. These fascinating gram-negative bacteria are able to undergo a developmental life cycle including the formation of multicellular "fruiting bodies" upon starvation. The largest known myxobacterial strain collection exists at the Helmholtz Centre for Infection Research with about 7500 isolates including novel moderately thermophilic myxobacteria described by Gerth and Müller [8]. This group of thermophilic myxobacteria grows between 30°C and 48°C, with a temperature optimum between 42°C and 44°C. In contrast, the temperature optimum for the growth of other myxobacteria is between 30°C and 34°C. Interestingly, moderately thermophilic myxobacteria grow faster than most other myxobacteria [8].

Most of the natural products produced by myxobacteria are polyketides, nonribosomally made peptides or hybrid compounds. The biosynthesis of these compounds is catalyzed by complex and multimodular polyketide synthases (PKS) or nonribosomal peptide synthetases (NRPS) comprising numerous domains which are responsible for each catalytic step in the corresponding biosyntheses starting from activated short chain carboxylic acids or amino acids [10]. To date, various PKS and NRPS biosynthetic gene clusters have been identified including several from myxobacteria, e.g. those directing the biosynthesis of the electron transport inhibitors myxothiazol and melithiazol [11,12], the potential anticancer agents epothilones and tubulysins [13-15], the myxochromides [16], disorazols [17], chivosazols [18], myxovirescins [19] and some other natural products with antibacterial, antifungal or cytotoxic activities [20]. The available genome sequences showed that in most cases the genome of the producer organism encodes more biosynthetic gene clusters than mirrored by identified compounds. Therefore, the genetic potential to produce secondary metabolites is higher than originally expected due to so-called "silent" genes [21]. Whether these genes are indeed "silent" or the amount of produced compound is too low for detection is a matter of debate. In a recent study, we could show that 11 out of 18 biosynthetic gene clusters in *M. xanthus *DK1622 are indeed expressed and translated into proteins during vegetative growth although only five compounds are known from this strain [19,21-24]. A similar situation is obvious for *Sorangium cellulosum *So ce56, which also contains more genes potentially involved in the production of the secondary metabolism than expected after the isolation of the natural products from the culture extracts [21,24-26].

One of the possibilities to explore the genetic potential of such microorganisms or to deliberately modify natural product biosynthesis is the heterologous expression of the corresponding biosynthetic gene clusters. This is particularly useful if the manipulation of the chromosome in the producer strain is difficult, as in many myxobacterial strains.

This method allows to access the biosynthetic genes even from metagenome libraries of unculturable microorganisms if appropriate heterologous hosts are chosen [27,28]. Therefore, the development of heterologous expression systems for the transfer of large biosynthetic gene clusters from the natural producer strain into more suitable and easily culturable heterologous hosts is of great significance for natural product research [29].

In this work, we characterize *Corallococcus macrosporus *GT-2 exemplarily for moderately thermophilic myxobacteria as heterologous hosts and describe the expression of the myxochromide megasynthetase based on a novel transposon gene cluster transfer strategy which also involved promoter exchange. Production of the natural product could be significantly increased from 8 mg/L in original producer *S. aurantiaca *to 600 mg/L in GT-2.

## Results and discussion

### Physiological properties of the isolate *C. macrosporus *GT-2

Thermophilic myxobacteria with potential for the biosynthesis of natural products were isolated and described for the first time in 2005 by Müller and Gerth [8]. The idea to use thermophilic myxobacteria as heterologous hosts for the expression of diverse myxobacterial biosynthetic gene clusters arose with the physiological characterization of this novel group of bacteria, which exhibits very useful features such as fast growth in combination with typical myxobacterial properties such as social behaviour.

Here, we characterize a new thermophilic isolate of *C. macrosporus *as model host organism. Strain GT-2 is a moderately thermophilic myxobacterium which grows in yellow coloured swarm colonies. Fruiting bodies resemble those of *Corallococcus *and the myxospores are spherical and optically refractile. The vegetative cells are slender rods with tapering ends as they are typical for members of the suborder Cystobacterineae. As published previously *C. macrosporus *has a separate systematic position and is more closely related to *M. xanthus *than to the other members of *Corallococcus *[30].

**Figure 1:**
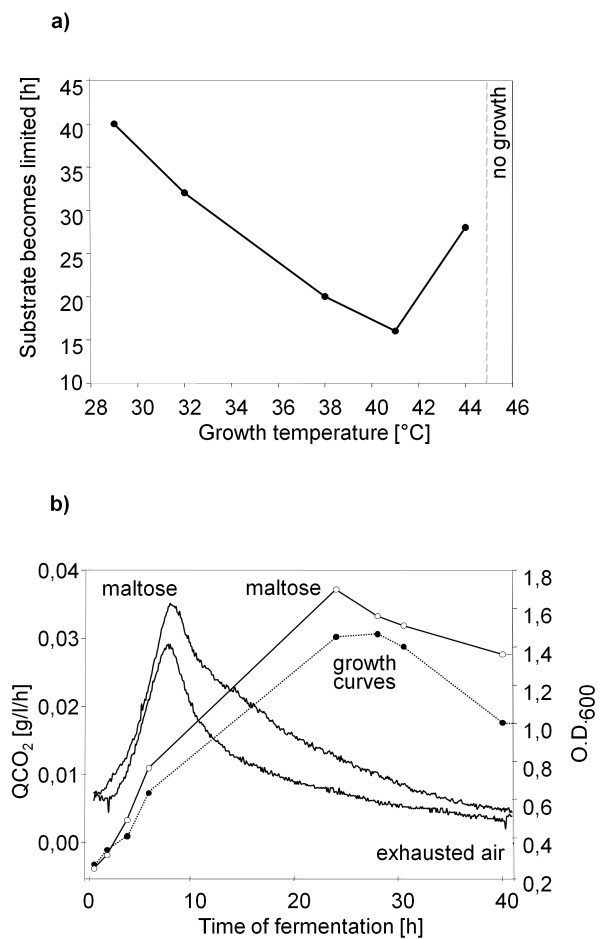
**a) The time required to reach substrate limitation is shown in dependence of the temperatures in 5 parallel fermentations**. The CO_2 _concentration in the exhausted air increased in dependency of the cell number as a consequence of metabolism of the carbon source. The faster the growth velocity, the earlier the carbon source became limiting resulting in a drop in CO_2 _concentration measured in the exhausted air. The period of time to reach this turning point is an indirect measure for growth velocity. b) Effect of maltose on growth and CO_2 _liberation compared to a control fermentation without alternative carbon source. The QCO_2 _was determined by balancing the exhausted air (see curves of exhausted air) as described. Growth was determined by measuring the optical density (black circle = control culture, open circle = culture with maltose added).

The 16S rDNA of GT-2 was sequenced and submitted in the NCBI BLAST basic local alignment search tool to identify similar sequences. The moderately thermophilic isolate is closely related to *C. macrosporus *(98% identity of the 16S rDNA to the type strain *C. macrosporus *Cc m8, DSM 14697) and was therefore named *C. macrosporus *GT-*2*. Like many nonadapted myxobacteria, *C. macrosporus *GT-2 initially grew in liquid medium in small cell aggregates. Therefore, the optimum growth temperature was determined in 15 l bioreactors by growing the bacteria at different temperatures (Fig. 1a). In the cause of the fermentations the carbon dioxide concentrations in the exhausted air were measured continuously (Fig. 1b). The time required to reach the turning points in carbon dioxide liberation (CO_2 _production declines because the carbon source becomes limiting) is easily monitored (Fig. 1b). This value can be used as a measure for growth velocity at the different temperatures (Fig. 1a).

With a temperature increase from 29°C to 41°C the time needed to reach substrate limitation decreased and increased again with a rise of temperature to 44°C, which was the highest temperature tolerated by this strain. The optimum temperature is approximately 42°C which is typical for moderately thermophilic myxobacteria. With a calculated generation time of little more than 2 hours this isolate grows three times faster than mesothermophilic *Myxococcus *isolates [8].

Growth and production of secondary metabolites of *Myxococcus *strains are limited by their restriction to amino acids as sole carbon and energy sources. Ammonium accumulates and becomes inhibitory to growth [3]. Little information is available for *Corallococcus *strains on alternative carbon sources. According to results published previously [30], *C. macrosporus *assimilates glucose. In our experiments, we could not verfiy this result for the strain under study. In contrast, maltose slightly stimulated growth of the GT-2 strain (Fig. 1b). During fermentation experiments in 15 l bioreactors, an effect of maltose was seen as an increase of the optical density, a higher maximum value in the produced CO_2 _and a slower drop during the following 30 hours of fermentation. In contrast to *Myxococcus xanthus*, GT-2 can use maltose as additional carbon source (under the given conditions about 7% consumption).

Salt concentrations of marine environments, about 3.2 to 3.8% of NaCl, inhibited the growth of GT-2 but concentrations up to 2% were well tolerated and resulted in doubling of the generation time to 4 hours. Salt tolerance may be a useful trait for heterologous expression of biosynthetic gene clusters from marine strains [27,31] because of possibly interconnected underlying regulatory processes.

Similar to most of the myxobacteria described before, *C. macrosporus *GT-2 was resistant against numerous antibiotics (Table 1) but the two commonly used selection markers kanamycin and oxytetracycline can be used representing an additional advantageous property of this strain.

**Table 1:** The inhibitory effect of 50 μg/ml of the respective antibiotic was determined by measuring the diameter of the swarm colony on agar plates incubated for 1 week at 30°C.

**Antibiotic**	**Diameter of the colony** **[mm]**
control	20

Ampicillin	12

Bacitracin	12

Cephalosporin	13

Fusidic acid	19

Gentamicin	15

Hygromycin	14

Kanamycin	0

Kasugamycin	7

Neomycin	11

Oxytetracycline	0

Polymyxin	11

Spectionmycin	15

Trimetoprim	6

Thiostrepton	21

### Development of a genetic manipulation system for GT2 and suitability of this strain as heterologous host

In order to utilize GT-2 as a heterologous host, a genetic manipulation system for this strain had to be developed. As marker gene, a kanamycin resistance cassette was chosen. Next, the possibility to integrate foreign DNA through homologous recombination was analyzed. As a target for homologous recombination a DNA fragment from the GT-2 chromosome was amplified using degenerate PCR primers targeting fragments of β-ketoacyl-ACP synthase (KS) domains of polyketide synthases [32]. The resulting DNA fragment was cloned into the pCR2.1TOPO^® ^cloning vector (Invitrogen) and sequenced to analyze the cloned PKS region. Subsequently, the resulting plasmid pOPB32 was introduced into the cells by electroporation. Different protocols were examined including the variation of such parameters as growth temperature of the cells prior to the preparation of competent cells, composition of washing and electroporation buffers, different voltage and pulse length as well as the incubation time after electroporation and the composition of the plating medium. Colony PCR was used as a method to screen for correct integrations of the suicide plasmid into the chromosome (data not shown).

Therefore, *C. macrosporus *GT-2 is the first strain from the collection of thermophilic myxobacteria for which a genetic manipulation system has been established.

Although no secondary metabolites could be isolated from GT-2 extracts derived from a set of growth conditions (including the variation of temperature; data not shown), the strain possesses genes encoding PKS which therefore seem to be "silent" under the tested laboratory conditions. This underscores the fact that the biosynthetic machinery for the biosynthesis of the heterologous product is available in the strain and at the same time the purification of the obtained product should be easier because it is not mixed with other produced metabolites.

Different techniques and strategies have been used for the heterologous expression with the aim to improve the yield of the natural product [29]. In general, the heterologous expression in phylogenetically related hosts is regarded most effective as they provide additional useful features such as similar GC content and a codon usage like in the donor strain. Also, other intracellular factors which influence heterologous expression [33] and affect stability of large transcripts, translational processivity of the long ORFs and protein folding are most likely available for the expression of biosynthetic genes in closely related strains. Another myxobacterium – *M. xanthus *– has already been used as a heterologous host for the biosynthesis of secondary metabolites from other myxobacterial species, e.g. epothilone from *S. cellulosum *[34], myxothiazol from *S. aurantiaca *[35] and myxochromide from *S. aurantiaca *[36]. However, GT-2 may in the future turn out to be an advantageous host for the reasons mentioned above and due to the fact that it grows two to three times faster than the well established *M. xanthus*.

### Integration of the myxochromide gene cluster into the chromosome of GT-2

**Figure 2:**
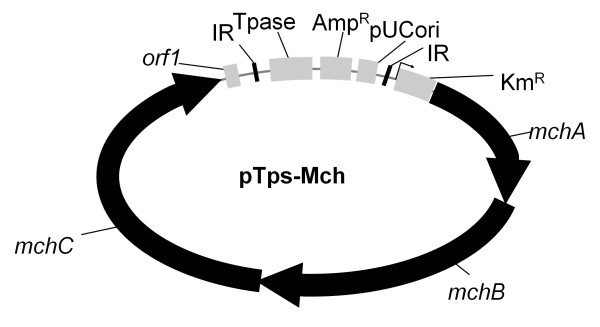
**Construct used for integration into the chromosome of *C. macrosporus *GT-2**. IR – inverted repeats; Tpase – transposase; pUC ori – origin of replication; Amp^R ^– ampicillin resistance selection marker; Km^R ^– kanamycin resistance selection marker; right-angled arrow (↱) – P_*aphII *_promoter; *mchA-mchC *– myxochromide biosynthetic genes; orf1 – gene with unknown function located adjacent to the *mch *genes (Fu et al., 2008).

The biosynthetic genes encoding the myxochromide S megasynthetase in *S. aurantiaca *DW4/3-1 have been cloned, sequenced and modified for heterologous expression in *Pseudomonas putida *previously [16,37]. Subsequently, a transposon containing the whole myxochromide biosynthetic gene cluster as transposable element was constructed [36]. The transposon harbouring the constitutive P_*aphII *_promoter driving the transcription of the myxochromide genes is shown in Figure 2. In contrast, the inducible Pm promoter was used to control expression in *P. putida *[37].

The integration of the plasmid pOPB32 into the chromosome of GT-2 followed by selection for the kanamycin resistance derived from the *aphII *gene verified that the promoter P_*aphII *_is also active in this strain.

**Figure 3:**
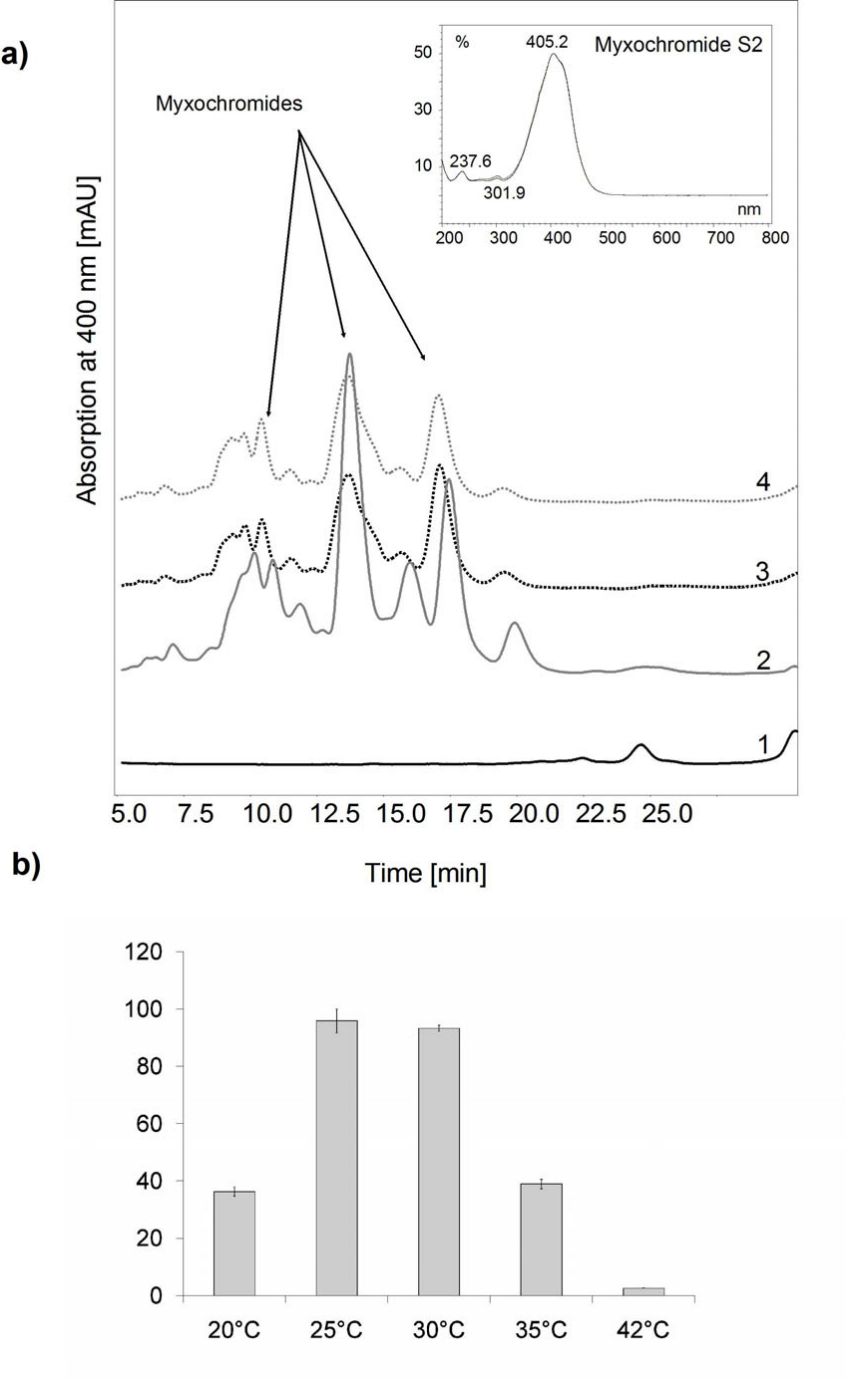
**Myxochromide production in *C. macrosporus *GT-2::mch a) HPLC-DAD (diode array detector) chromatogram: 1 – extract of *C. macrosporus *GT-2; 2 – 4 – extracts of mutant strains containing the myxochromide gene cluster integrated into the chromosome of GT-2**. Inset panel: UV spectrum of myxochromide S at 400 nm. b) Influence of the temperature on the heterologous myxochromide production in *C. macrosporus *GT-2::mch.

After electroporation kanamycin resistant colonies were obtained on the agar plates 2–3 days after incubation at 37°C. Ten randomly chosen colonies were analyzed by colony PCR (s. Experimental procedures). PCR analysis showed amplificates of the expected size of 593 bp and 700 bp, respectively and thus verified the successful transposition and integration of the myxochromide gene cluster into the chromosome (data not shown). As expected, the mutants containing the myxochromid biosynthetic gene cluster under the control of P_*aphII *_promoter produced the corresponding compound myxochromide S plus its derivatives constitutively (Fig. 3, 4.).

**Figure 4:**
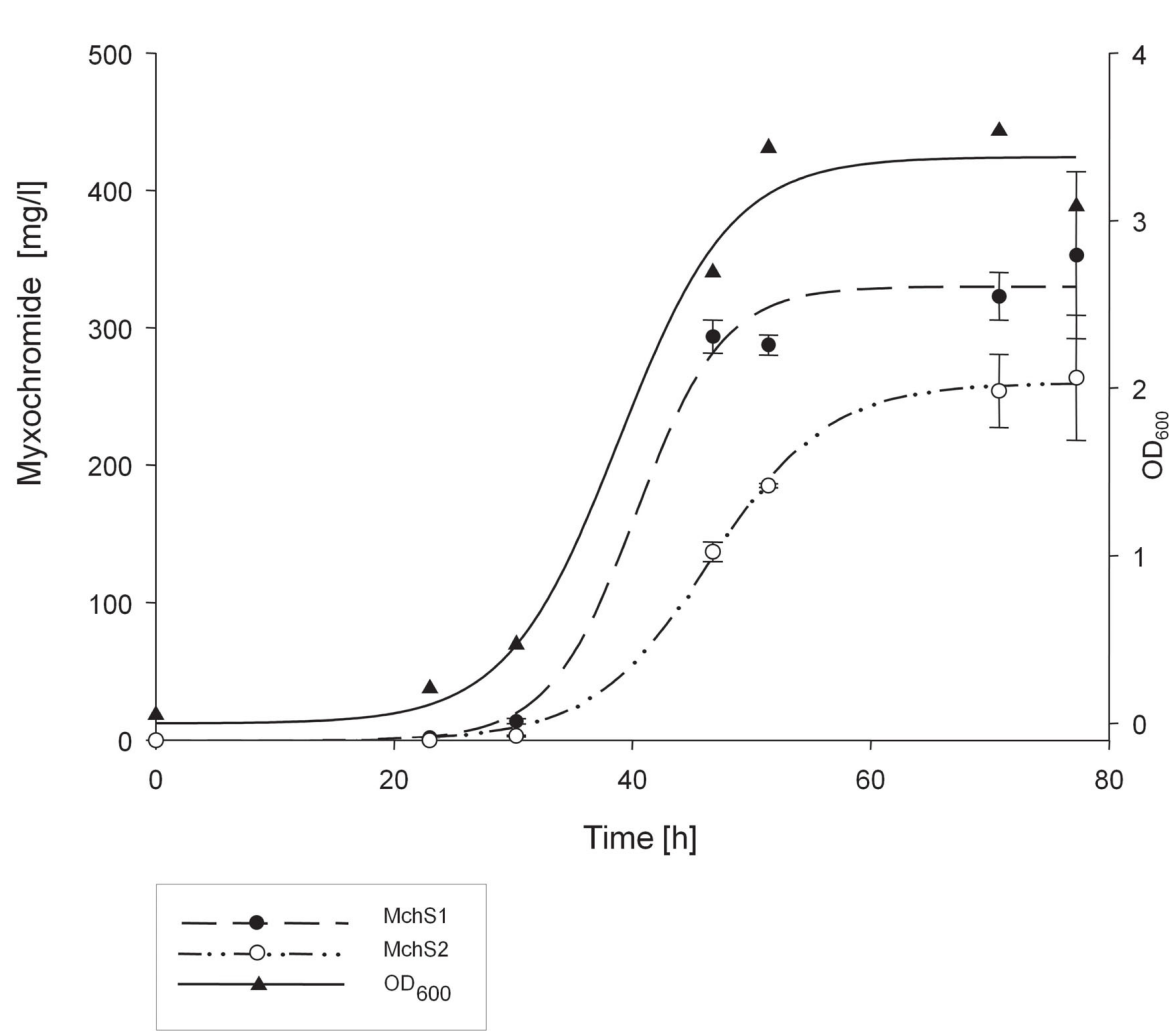
**Production kinetics of myxochromides S1 and S2 in *C. macrosporus *GT-2::mch at 30°C**.

### Analysis of the myxochromide production

Culture extracts of the mutant strains were investigated by HPLC analysis after growth at 30°C. It was possible to identify myxochromides S1-3 synthesized by the gene products of the cloned *mch *gene cluster from *S. aurantiaca *as well as additional myxochromide derivatives (Fig. 3a). Myxochromide S3 could be detected only in traces and could not be used for the quantification of total production yields. Additional peaks may represent derivatives of S1 and S2 lacking methyl groups as has been assumed also for the novel heterologous myxochromide derivatives in *P. putida *[37].

HPLC analysis showed that the tested mutants, which contain *mch *genes in the chromosome, differed in their ability to produce myxochromides relative to each other. The analysis of the integration position of the transposable element showed that the myxochromide biosynthetic genes, as expected, have been integrated into different chromosomal loci of the GT-2 genome (Table 2). The mutant strain producing the highest amount of myxochromide in comparison to other mutants was named *C. macrosporus *GT-2::mch and used for further studies.

**Table 2:** Integration sites of myxochromide biosynthetic genes in *C. macrosporus *GT-2.

	DNA sequence		
Mutant clone	IR (underlined) insertion site (bold italic)	Gene product	Organism, Identity, %

GT2::mch	---AGCGTGAAGACCGGGGACTTATCAGCCAACCTGT***TA*** GTTGGAGAGCGAGACGACGACGAGCT---	NADP oxidoreductase	*Myxococcus xanthus *DK1622, 91

P1	---AGCGTGAAGACCGGGGACTTATCAGCCAACCTGT***TA*** CACCTGTGCAGCCGGGCTCACCCCGG---	Hypothetical protein	*Myxococcus xanthus *DK1622, 79

P2	---AGCGTGAAGACCGGGGACTTATCAGCCAACCTGT***TA*** GATGGCGCTGACGCGAATGATGACCT---	site-specific recombinase, phage integrase family protein	*Mycobacterium avium *104, 31

P3	---AGCGTGAAGACCGGGGACTTATCAGCCAACCTGT***TA*** GCCCAGGCCCGCTTCACCGCCAATGG---	Predicted transcriptional regulator	*Magnetospirillum magneticum* AMB-1, 60

P5	---AGCGTGAAGACCGGGGACTTATCAGCCAACCTGT***TA*** GTCATGGACCTCCGTGACGGTGCCCA---	excinuclease ABC, A subunit	*Myxococcus xanthus *DK1622, 98

P6	---AGCGTGAAGACCGGGGACTTATCAGCCAACCTGT***TA*** CGTGCCCATCGACGCCTCACCGGCGA---	hydrolase, CocE/NonD family	*Myxococcus xanthus *DK1622, 72

In order to find optimal conditions for the heterologous myxochromide production first the influence of the temperature on the production was examined. The cultures were incubated at different temperatures from 20°C to 42°C for 3 days and the production of myxochromides was compared. Interestingly, almost no production could be detected in cultures which were grown at 42°C. While the optimal growth temperature for GT-2 is 42°C (Fig. 1a), the most favourable temperature for heterologous myxochromide production was found to be 25–30°C (Fig. 3b).

Although it could already be shown for other secondary metabolite producers that product formation is dependent on temperature and in many cases increases with lower temperatures even in natural producer strains, e.g. phaseolotoxin production by *Pseudomonas syringae *pv. phaseolicola [38] or phenazine production by *P. fluorescens *2–79 [39], the highest level of production of different secondary metabolites in thermophilic myxobacteria, e.g. myxothiazols, phenalamids and stigmatellins, has been detected at the optimal growth temperature of 42°C [8]. For the heterologous biosynthesis of myxochromides in GT-2 the expression of foreign proteins (PKS and NRPS) itself might be stressful and lower temperatures might reduce protein misfolding by slowing down transcription and translation rates, especially in cases when the proteins are expressed from strong promoters (such as P_*aphII *_in our case) as has been described in *E. coli *[40,41]. Interestingly, the expression of the kanamycin resistance gene occurred even at higher temperature and allowed the selection of the kanamycin resistant mutants already after 2 days of growth on the agar plates. This is highly advantageous because *M. xanthus*, another heterologous expression host, requires 5–10 days for the growth on selection plates.

Kinetic studies of myxochromide production were carried out at 30°C. The production correlated with the number of cells in the culture, so the maximum of production and a constant amount of the product was reached with the stationary phase of the culture. The production maximum was reached after 2 days, compared to 6 days required when using *S. aurantiaca*. GT-2 produced approximately 350 mg/L myxochromide S1 and 250 mg/L myxochromide S2 (Fig. 4). The product yield of just the major compounds S1 and S2 was thus larger than 600 mg/L and therefore much higher than in the original producer *S. aurantiaca *(8 mg/L total) and in the previously described heterologous host *P. putida *(40 mg/L total) [37] although in recently described studies of heterologous production of myxochromides in *M. xanthus *production of about 1 g/L could be shown [36].

### Transposon based integration of secondary metabolite biosynthetic genes allows screening for alternative heterologous hosts

Recently, we described the advantages of Red/ET recombineering for the generation of constructs for the integration into heterologous hosts [35,37,42]. For the expression of the myxothiazol gene cluster the construct containing homologous regions for integration either into *M. xanthus *or *P. putida *was introduced into the vector containing all myxothiazol biosynthetic genes. The disadvantage of this strategy is the requirement of the introduction of different homologous chromosomal targets for each heterologous host strain. In this study, we used a transposon based strategy which can be employed in a very flexible way for the introduction of the same construct into different heterologous hosts in parallel experiments to investigate the suitability of different hosts and conditions for metabolite production.

With the described transposon based strategy the myxochromide biosynthetic genes were successfully integrated into already described heterologous host strains – *M. xanthus *and *P. putida *[36].

The approach described here might be especially useful in the future for the expression of silent biosynthetic gene clusters [21] which can be activated by inserting promoters constitutively active in a variety of host strains. Moreover, our studies showed that *C. macrosporus *GT-2 may be developed into a very useful heterologous host for myxobacterial secondary metabolite production in the future.

## Methods

### Isolation of GT-2

The method for the isolation of moderately thermophilic myxobacteria has been described previously in detail [8] GT-2 was isolated from a soil sample from Sousse (Tunesia).

### Media (constituents in g/l) and maintainance of liquid cultures

Medium M: Soy peptone (Marcor), 10; Maltose; 10: CaCl_2 _× 2 H_2_O, 1; MgSO_4 _× 7 H_2_O, 1; HEPES, 11.9; Fe-EDTA, 8 mg; pH adjusted to 7.2.

Basic fermentation medium: Soy peptone (Marcor), 5; CaCl_2 _× 2 H_2_O, 1; MgSO_4 _× 7 H_2_O, 1; Fe-EDTA, 8 mg; ZnCl_2_, 1 mg; K_2_HPO_4 _0,1 g; pH adjusted to 7.2.

Liquid cultures were started by inoculating 100 ml of medium M in 250 ml erlenmeyer flasks with 5 ml of precultures. Inoculated flasks were incubated at 42°C on a rotary shaker at 160 rpm for 4 days.

### Fermentation conditions

Basic fermentation medium: Soy peptone (Marcor), 5; CaCl_2 _× 2 H_2_O, 1; MgSO_4 _× 7 H_2_O, 1; Fe-EDTA, 8 mg; ZnCl_2_, 1 mg; K_2_HPO_4 _0,1 g; pH adjusted to 7.2.

Fermentations were performed in 15 l bioreactors (Fermenter B10, Fa. Biologische Verfahrenstechnik) with 10 l of fermentation medium inoculated with 1 l of a preculture grown under shaking (160 rpm) in the same medium. The fermentations were run at 29°C, 32°C, 38°C, 41°C, 44°C and at 45°C with an aeration of 0.1 vvm (volumes of air per minute per volume of batch culture). A pH of 7.0 was maintained by the addition of 5% sulphuric acid.

Dissolved oxygen was measured with a polarographic stream sterilizable dissolved oxgygen electrode (Ingold). A pO_2 _of 40% was maintained by regulation of the stirrer speed. The exhausted air was measured in a continuous gas analyser (H. Maihak AG, Hamburg equipped with unor -CO_2 _analyser and oxor -oxygen analyser).

### Test for effects of carbohydrates on growth

The effect of maltose on growth was investigated in the basic fermentation medium with additional 10 g/l of maltose and 0.25 g/l of ammonium sulfate. Growth at 42°C was followed by measuring of the optical density (O.D.) at 600 nm and the carbon dioxide production.

### Calculation of the generation time

Growth in medium M at 42°C was analyzed every 2 hours by counting the cell number in a counting cell (Neubauer improved, 0,02 mm depth; 0,0025 mm^2^). The generation time was calculated by the following equation: g = lg2 × t/lgN_1 _- lgN_0 _(g = generation time; t = time of incubation; N is the mean value of the cell number at time 0 and time 1)

### Growth in the presence of NaCl

The effect of sodium chloride was investigated in 100 ml medium M in 250 ml Erlenmeyer flasks in the presence of increasing concentrations of NaCl. For the determination of the generation time the following medium was used: marine broth (Difco), 37.4 g/l; peptone (Marcor typ S), 5 g/l; HEPES buffer, 100 mM, pH 7.4.

### Antibiotic resistance

After autoclaving of the agar medium M, antibiotics were added to a final concentration of 50 μg/ml. The plates were incubated at 30°C. After 1 week the diameter of the swarm colonies was measured as an indication of growth.

### Analysis of maltose content

Culture broths of the fermentations were analysed twice a day for the maltose content. After centrifugation for 30 min at 10.000 rpm in a Sorvall cool centrifuge, 20 μl of the supernatant was mixed with 177 μl of distilled water and 3 μl of a α-glucosidase solution. After incubation for 1 h at 37°C the reaction solution was incubated at 80°C for 15 min and centrifuged again. The supernatant was diluted 1:10 with distilled water and the glucose concentration was analysed with a hexokinase test kit (Boehringer Mannheim).

### Sequencing of the 16S rDNA from GT-2

A fragment was generated by PCR (Taq DNA polymerase), using genomic DNA as template and the primers SB19 and SB20 [43] The PCR fragment was cloned using the TOPO TA cloning kit (Invitrogen). The insert of the resulting plasmid was sequenced and compared with the NCBI data bank.

### Electroporation into *C. macrosporus *GT-2

The GT-2 cells were grown in liquid medium M at 30°C to a final cell density of 10^9 ^cells/ml. After centrifugation and washing with washing buffer (1 mM HEPES, 0.5% glucose, pH 7.4), cells were resuspended in 1 ml of electroporation medium (0.5% glucose in distilled H_2_O) to 10^10 ^cells/ml. The cell suspension (100 μl) was mixed with up to 1 μg of DNA and electroporated (BioRad electroporator) at 25 μF, 1200 V (τ = 5 ms) using 0.1 cm cuvette. After electroporation 900 μl M medium was added, then the cells were resuspended in 10 ml M medium and the Erlenmeyer flasks were incubated at 37°C or at 42°C on a rotary shaker at 160 rpm overnight. After centrifugation the cells were resuspended in 1 ml liquid M medium and different cell dilutions were plated for selection on M agar plates (medium M with 1.5% agar) supplemented with 50 μg/ml kanamycin. The kanamycin resistant colonies arising after 2 – 3 days at 42°C were tested for growth in liquid medium. Integration of the marker gene was verified by colony PCR with different primers specific for the integrated DNA.

### Analysis of the integration of the myxochromide biosynthetic gene cluster

The transposon used in this study is shown in Figure 2[36]. After electroporation the mutants were analyzed by colony PCR for the integration of the myxochromide biosynthetic gene cluster into the chromosome using two primer pairs: Mch35 and Mch36 [37], and Mch3 (5'-CAAGACGGCCACAGTGAGTCGATGAC) and Mch4 (5'-CAAGCGCGCGAAGCGCCTCGGGGC) amplifying 700 bp and 593 bp fragments of the myxochromide biosynthetic genes, respectively. The mutant strain containing the myxochromide biosynthetic gene cluster and producing highest amount of myxochromide in comparison to other mutants was named *C. macrosporus *GT-2::mch and used for further studies.

In order to identify the insertion sites of the *mch *gene cluster in GT-2 strain the chromosomal DNA from 6 different mutants was analyzed. For this purpose a two-step procedure has been applied. The first step was a single-primer PCR (5'-gcaattccggttcgcttgct) based on the rapid amplification of transposon ends (RATE) protocol [44]. For the second step – sequencing – the additional nested primer (5'-ccagtagctgacattcatcc) was used. The preceeding PCR was performed as a three-step, single-primer PCR. The first and third steps were performed at stringent temperature whereas the second step was performed at low temperature (30°C). After initial denaturation for 5 min at 95°C 30 cycles of amplification were performed: 30 s at 95°C, 30 s at 55°C and 30 s at 72°C. The second step included 30 cycles: 30 s at 95°C, 30 s at 30°C and 30 s at 72°C. The last step included 30 cycles as in first step followed by a final extension at 72°C for 10 min. In the first step the single-stranded products were generated, which were then amplified in a second step at low temperature with non-specific binding of the same primer. The third step amplified specific and non-specific products at stringent temperature. PCR reactions were purified and sequenced with specific nested primers. The results are shown in Table 2.

### HPLC-MS analysis of myxochromide production

Fifty ml medium M in a 300 ml flask was inoculated with 2.5 ml of an overnight culture of strain GT-2::mch and XAD 16 (Rohm und Haas, Frankfurt, 2%) was added routinely. After 3 days of incubation with shaking at the temperatures given in *Results and discussion *the cells were harvested by centrifugation and extracted with acetone and methanol. The extracts were evaporated and dissolved in 1 ml methanol. 5 μl of the extracts were analyzed by HPLC (Dionex solvent delivery system coupled to PDA-100 Photodiode Array Detector).

The detection was carried out at 400 nm. For quantification the extracts were prepared from 1 ml culture and analyzed by LC-MS (Agilent 1100 series, Bruker HCT plus Ion Trap mass spectrometer). The chromatographic conditions used were: RP column Nucleodur C18 (Macherey-Nagel), 125 × 2 mm, 3 μm, and precolumn C18, 8 × 3 mm, 5 μm. A solvent gradient (using solvent A and B with solvent A being water plus 0.1% formic acid, and solvent B being acetonitrile plus 0.1% formic acid) from 60% B at 1 min to 95% B within 6 min was applied and followed by 2 min at 95% B. The myxochromides were identified by comparison to the retention times and the MS fragmentation pattern of the authentic reference substances (parent ions are given: myxochromide S1: [M+H]^+ ^= 723; myxochromide S2: [M+H]^+ ^= 737; myxochromide S3: [M+H]^+ ^= 749).

## Abbreviations

IR: inverted repeat; kbp: kilobase pair; ORF: open reading frame.

## Competing interests

The authors declare that they have no competing interests.

## Authors' contributions

OP transformed GT-2, analyzed the production kinetics and drafted the manuscript. KG isolated and characterized GT-2 and participated in writing of the manuscript. SK participated in the molecular biological studies. YZ participated in the design of the study. RM coordinated the study and wrote the manuscript. All authors read and approved the final manuscript.
